# The Unwise Preacher

**Published:** 1874-01

**Authors:** 


### THE UNWISE PREACHER.

He had been a preacher forty-one years, never missing a single Sunday; but he was only sixty-seven, and resigned his charge because he was so old! I am afraid that pride was at the bottom of that thing, which the adversary made a handle of to get out of the pulpit—one of the most useful and efficient preachers that ever went into it. He preferred to retire with flying colors. He chose rather to walk out than wait a little longer and run the risk of being kicked out.

We like rather the sturdy course of that old Baptist clergyman who preached away until he was eighty, and on being requested to retire, or have an assistant, answered: "What do I want with an assistant? I can preach as well as I ever could." There he "stuck," and would not move a peg.

The truth is, there is a great mistake about disposing of gray-headed men, sending them out to grass and bringing in young fellows to "grow up with the church;" there are several such in and around New York to-day, and to our own personal knowledge they have not grown a speck these five years. One of them grew so rapidly in the direction of a cow's tail, and the church he had charge of, too, that the people waked up one day, and in a fit of desperation sent him off packing. There are still two or three sticks in and near Fifth Avenue, who are nothing less than "cumberers of the ground," and they had better clear out; the trustees know this, but are afraid to "speak up in meeting." The older a lawyer gets, or a physician, the better are they, with all their experience, and observation, and reading, and thought. A man's mind is not old at sixty-seven, even if his body is; it is about that time the immortal part is beginning to blossom anew for its immortality. With a wise, with a reasonable attention to the laws of health, a professional man may be a vigorous worker and thinker until fourscore. As to clergymen particularly, what experience have they gathered, with what carefulness do they adopt new things, with what affection do they cling to the "old paths," and what stores of prudence, and forbearance, and patience have they laid up, and how, like the Master, have they learned to "make allowance" for the failings of men, having been, all along their journey through, tempted in all points as they were, and hence can be "touched with the feeling of the infirmities" of the weak, and frail, and tempted. They do not go off with a flash, and by harsh words and harsh deeds discourage the erring down to the bottomless pit. One word for us, stick to the old preachers, "WHICH IS FAR BETTER."

